# Pyriproxyfen Disrupts Chitin and Trehalose Metabolism in the Silkworm *Bombyx mori*

**DOI:** 10.3390/insects17030301

**Published:** 2026-03-11

**Authors:** Yizhou Zhu, Yuting Wei, Zhenfeng Zhou, Yizhe Li, Kaizun Xu

**Affiliations:** 1 College of Agriculture, Guangxi University, Nanning 530004, China; 19177551725@163.com (Y.Z.); weinnning@163.com (Y.W.); 15079697628@163.com (Z.Z.); ppsxj003@163.com (Y.L.); 2 Guangxi Key Laboratory of Agro-Environment and Agric-Products Safety, National Demonstration Center for Experimental Plant Science Education, College of Agriculture, Guangxi University, Nanning 530004, China

**Keywords:** *Bombyx mori*, pyriproxyfen, chitin, trehalose, gene expression

## Abstract

Pyriproxyfen, a juvenile hormone analog insecticide, poses severe risks to non-target silkworms (*Bombyx mori*), as evidenced by disrupted metamorphosis—a process strictly dependent on chitin synthesis and its precursor trehalose. However, the specific molecular interference of pyriproxyfen in these metabolic pathways remains unclear. This study investigated the transcriptional response of silkworm midguts to pyriproxyfen using RNA-Seq and validated spatiotemporal gene expression via qRT-PCR. By integrating transcriptomic data with long-term spatiotemporal profiling, we revealed novel tissue-specific expression dynamics. RNA-Seq identified 2059 differentially expressed genes, primarily enriched in metabolic pathways. Spatiotemporal analysis revealed that most chitin- and trehalose-related genes generally exhibited a biphasic “suppression–compensation” trend (initial downregulation followed by upregulation). Notably, tissue-specific responses were evident, with *ChsA* being continuously suppressed in the middle silk gland, which may be associated with impaired sericin secretion, while showing abnormal upregulation in the posterior silk gland. Additionally, trehalose metabolism genes (*Treh* and *Tret*) paralleled the fluctuation of chitin genes, indicating systemic metabolic reprogramming. These results suggest that the toxicity of pyriproxyfen is associated with a decoupling of trehalose metabolism from chitin synthesis and the induction of tissue-specific metabolic disorders. The tissue-specific, long-term spatiotemporal profiling of chitin and trehalose genes presented in this study fills a critical knowledge gap. This study characterizes the transcriptional profile associated with pyriproxyfen toxicity and provides a robust molecular reference for assessing its environmental risks to beneficial insects.

## Simple Summary

Pyriproxyfen is a pesticide widely used to control agricultural pests, but it also poses risks to beneficial non-target insects like the silkworm. While this chemical is known to damage silk production and prevent silkworms from maturing, the specific biological reasons for this toxicity remain unclear. This study investigated the transcriptional basis of how pyriproxyfen exposure is linked to altered metabolism of two vital substances: chitin, the material that forms the insect’s hard outer skeleton and internal structures, and trehalose, the primary blood sugar used as energy to build chitin. By analyzing gene activity in silkworms exposed to the pesticide, this research aimed to characterize the transcriptional response associated with pyriproxyfen toxicity. The exposure was associated with a chaotic fluctuation in gene levels, coinciding with the suppression of biological functions and triggering an ineffective repair attempt. These transcriptional changes are consistent with the disrupted silk gland development and larval–pupal metamorphosis observed previously. These findings are crucial for understanding the hidden dangers of pesticide residues in the environment. Furthermore, this research provides a scientific basis for assessing environmental risks and developing safer, more selective pest control strategies. The data also suggest a potential compensatory metabolic response under sustained insecticide exposure that protects economically important insects.

## 1. Introduction

Pyriproxyfen is characterized by high efficiency, low application rate, long persistence, safety to crops, and minimal impact on the ecological environment. It is mainly used to control pests belonging to Thysanoptera [1], Diptera [2], Lepidoptera [3], and Hemiptera [4]. However, pyriproxyfen can also cause adverse effects on some non-target organisms. It has been reported that pyriproxyfen is toxic to beneficial insects such as lacewings, honeybees, and silkworms. For instance, it can lead to epithelial folding, cytoplasmic protrusions, vacuolation of trophocytes in the fat body region, and mitochondrial damage in *Ceraeochrysa claveri* [5]. In addition, pyriproxyfen can interfere with the social behavior of honeybees and cause wing atrophy in adult bees [6]. In silkworms, even trace amounts of pyriproxyfen can induce adverse reactions, leading to silk gland damage, reduced silk production, and a decreased cocooning rate, among other effects [7].

Chitin, a ubiquitous biopolymer in nature, is primarily derived from the shells of crustaceans and the cell walls of fungi [8,9]. As a vital structural component of insect exoskeleton, silk gland ducts, and midgut peritrophic matrix (PM), the synthesis and degradation of chitin are critical processes for maintaining normal insect physiological functions [10,11,12].

The biosynthesis of chitin is regulated by chitin synthases (CHS), which are generally categorized into two classes: *ChsA* and *ChsB* [13]. The *ChsA* gene is expressed throughout the developmental stages of silkworms and is primarily responsible for catalyzing chitin synthesis in the exoskeleton [14,15]. Conversely, Chitin synthase B is a specifically functional protein encoded by the *ChsB* gene, and it mediates the chitin biosynthesis in the midgut peritrophic membrane of insects [16,17]. Previous studies have shown that the mortality rate of *Spodoptera exigua* (Lepidoptera: Noctuidae) was significantly increased when treated with lufenuron after *ChsA* silencing. It indicates that chitin synthase genes play a critical role in insect development [18].

In the degradation pathway, chitin deacetylases (CDA) initiate chitin decomposition [19,20]. Specifically, *CDA1* participates in the structural modification process of insect epidermal chitin and represents a potential molecular target for novel insecticides [21].

Chitinases (Chts) play a crucial role during insect molting and are closely associated with chitin decomposition [22,23]. Within this family, *Cht10* is highly expressed during the larval stage, and its inhibition results in abnormal molting and increased mortality in *Tetranychus urticae* [24].

Furthermore, members of the Mucin family are integral structural components of the peritrophic matrix (PM) [25]. Studies indicate that Mucin-like proteins not only localize to the PM but also serve as important salivary components that contribute to virulence, host adaptation, and immunity in the brown planthopper (*Nilaparvata lugens*) [26,27,28]. Additionally, the *Mucin* family gene *NlMuc2* has been shown to be closely associated with early embryonic development in *Nilaparvata lugens* [29], whereas in *Bombyx mori*, the family member *Mucin91C* is linked to tracheal development [30]. Despite the diverse functions identified within this family, the specific biological roles of *Mucin-2-X1* and *Mucin-2-X2* in *Bombyx mori* remain uncharacterized.

Insect chitin biosynthesis is a complex biochemical process in which the primary precursor is derived from trehalose. Trehalose, a non-reducing disaccharide composed of two glucose molecules, serves as the principal blood sugar in insects. It functions not only as an energy reserve but also as a key stress protectant under adverse conditions [31,32]. Intracellularly synthesized trehalose is mainly transported across cell membranes into the hemolymph via the trehalose transporter (Tret) and delivered to various tissues [33]. Trehalase (Tre) is the key enzyme regulating trehalose metabolism and includes two main types: soluble trehalase (*TRE1*) and membrane-bound trehalase (*TRE2*) [34,35]. Both enzymes are present in the midgut, indicating that the midgut is a critical tissue for studying trehalose metabolism and the supply of chitin synthesis precursors. As enzymes involved in insect energy metabolism and the chitin synthesis pathway, their gene expression is closely associated with vital physiological processes such as development, molting, metamorphosis, and reproduction [36].

Sericulture is a traditional advantageous industry in China; however, pesticide drift and contamination cause severe poisoning in silkworms, restricting industrial development and resulting in significant economic losses. *Bombyx mori* is not only an important economic insect but also a model organism for studies in toxicology and other fields. Also it is known that pyriproxyfen reduces the pupation rate and increases pupal mortality in silkworms [37], and that silk gland development and metamorphosis are highly dependent on normal chitin synthesis [38,39]. However, the molecular basis linking pyriproxyfen exposure to these developmental deficits remains poorly characterized. We hypothesized that pyriproxyfen exposure triggers tissue-specific transcriptional alterations in the chitin-trehalose metabolic axis, which are associated with the physiological defects observed in non-target insects. To address this previously diffuse biological question, the major aim of this paper is to map the specific molecular responses of the coupled chitin-trehalose axis under pesticide stress. Therefore, this study aims to test this hypothesis by integrating transcriptomic data with comprehensive spatiotemporal profiling. The transcriptomic insights from this study offer a basis for further investigation into the sublethal toxicity of pyriproxyfen on silkworms and may inform the design of more selective insecticides based on the chitin synthesis pathway.

## 2. Materials and Methods

### 2.1. Animals and Chemicals

The silkworm larvae strain (932 · Furong × 7532 · Xianghui) used in this study was reared at the Institute of Sericulture, Guangxi University, China. The larvae were maintained at 25 ± 1 °C, with a 12:12 (L:D) h photoperiod and fed with fresh mulberry leaves.

The pyriproxyfen (2-[1-methyl-2-(4-phenoxyphenoxy) ethoxy] pyridine, CAS NO.: 95737-68-1, purity ≥ 99.5%) was purchased from ANPEL Laboratory Technologies Inc. (Shanghai, China). A stock solution (1000 mg/L) was prepared in methanol and further diluted to a working concentration of 100 ng/L according to our previously described method [40,41].

RNAiso Plus and cDNA synthesis kit were purchased from TaKaRa (Dalian, China). ChamQ Universal SYBR qPCR Master Mix was purchased from Vazyme Biotech Co., Ltd. (Nanjing, China).

### 2.2. Sample Preparation

Previous studies have shown that pyriproxyfen is toxic to fifth-instar silkworms at concentrations of 10^−5^–10^−4^ mg/L, while it prevents cocooning at 10^−3^ mg/L [42]. Therefore, a sublethal concentration of 100 ng/L was chosen for this experiment to investigate its sublethal effects.

On the first day of the fifth instar, larvae were fed three times for 24 h with mulberry leaves treated with pyriproxyfen (treatment group) or the solvent (control group). They were then provided with fresh leaves until cocooning, as described previously [40].

At 24 h intervals post-exposure, enough silkworms were randomly sampled to dissect the epidermis, anterior silk gland, middle silk gland, posterior silk gland and midgut on ice. All tissues were immediately frozen in liquid nitrogen and stored at −80 °C for subsequent analysis.

### 2.3. Total RNA Isolation and cDNA Synthesis

Total RNA was isolated from tissues (epidermis, anterior silk gland, middle silk gland, posterior silk gland and midgut) with RNAiso Plus reagent (TaKaRa). Following extraction, genomic DNA was removed using a DNase treatment kit (TaKaRa). cDNA was then synthesized from the DNase-treated RNA using M-MLV Reverse Transcriptase and an oligo(dT) primer (TaKaRa).

### 2.4. Preparation and Sequencing of Digital Gene Expression (DGE) Library

Midguts from the control and treatment groups were dissected at 24 h post-exposure by pyriproxyfen for transcriptome analysis. The clean reads were aligned to the silkworm genome (https://silkdb.bioinfotoolkits.net/main) (access date: 16 December 2025) using HISAT2 [43].

Differential expression analysis identified significant differentially expressed genes (DEGs) (fold change ≥ 2 and Q-value ≤ 0.05), which were subsequently subjected to KEGG pathway enrichment analysis.

### 2.5. Real-Time Fluorescence Quantitative PCR (qRT-PCR) Analysis

All selected gene sequences were obtained from NCBI (https://www.ncbi.nlm.nih.gov/). qRT-PCR primers (Table 1) were designed at the NCBI website and synthesized by Beijing Tsingke Biotech Co., Ltd. (Beijing, China). The *Bombyx mori Actin3* gene was selected as an internal reference gene. qRT-PCR reaction was performed using LightCycler^®^ 96 System (Roche, Basel, Switzerland) with ChamQ Universal SYBR qPCR Master Mix (Vazyme Biotech Co., Ltd., China) according to the manufacturer’s instructions.

### 2.6. Data Processing

Statistical analyses were conducted with IBM SPSS Statistics 27. The effects of different treatments and time intervals, as well as their interactions, were determined by two-way ANOVA. Independent samples *t*-tests were employed to identify significant differences between groups at each time point. All results are expressed as mean ± SD from three independent biological replicates. Figures were processed using GraphPad Prism 9.5.1 software. The threshold for statistical significance was defined as *p* < 0.05.

## 3. Results

### 3.1. Transcriptomic Analysis of the Silkworm Midgut Following Pyriproxyfen Exposure

**Figure 1:**
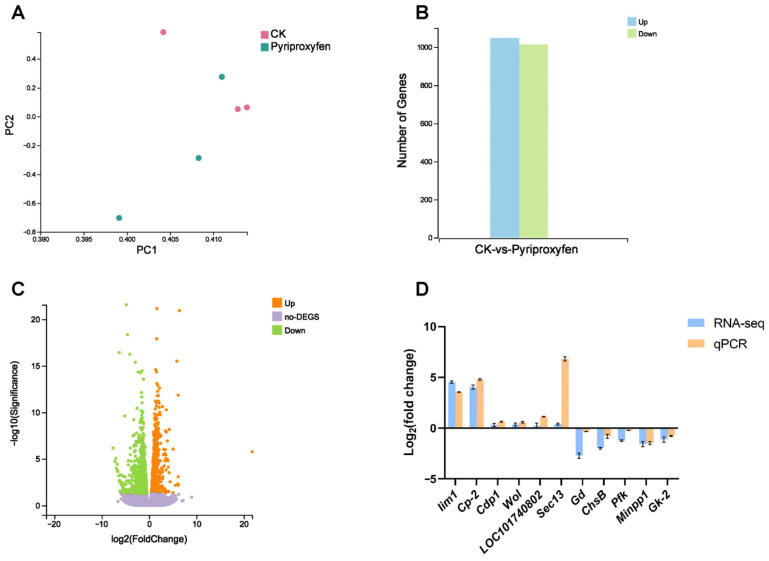
Transcriptomic analysis and qRT-PCR verification 24 h following pyriproxyfen exposure. (**A**) Principal Component Analysis (PCA) of control and treatment groups. (**B**) Statistical summary of upregulated and downregulated DEGs. (**C**) Volcano plot distribution of DEGs between the control and treatment groups. (**D**) qRT-PCR validation of genes related to chitin and carbohydrate metabolism. Data are presented as mean ± SD (n = 3). *Cp-2*: chondroitin proteoglycan-2 gene; *Cdp1*: chitinase domain-containing protein 1 gene; *Gd*: glucose dehydrogenase gene; *Minpp1*: multiple inositol polyphosphate gene.

Our previous studies demonstrated that pyriproxyfen is associated with disrupted silk gland development and failed larval–pupal metamorphosis in silkworms [39,40,41]. In silkworms, chitin not only exists in the larval epidermis but also forms the cuticular intima of the silk gland together with cuticular proteins, which exerts an important impact on the structure and properties of silk. The midgut is not only a site for chitin synthesis but also the main tissue for trehalose utilization. Therefore, we conducted RNA-seq on midgut tissues 24 h after pyriproxyfen exposure. Principal Component Analysis (PCA) revealed a distinct separation between the control and treatment groups (Figure 1A). A total of 2059 DEGs were identified, comprising 1046 upregulated and 1013 downregulated genes (Figure 1B,C). To validate the RNA-seq sequencing data, eleven genes involved in chitin and carbohydrate metabolism were randomly selected for qRT-PCR analysis. The results showed that the expression patterns of these genes were consistent with the RNA-seq results (Figure 1D), indicating that the RNA-seq results are reliable.

### 3.2. GO and KEGG Enrichment Analysis of DEGs

**Figure 2:**
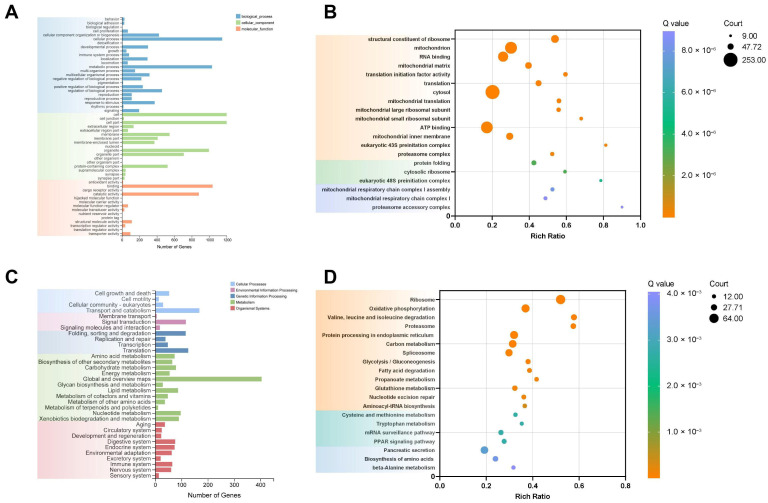
Gene Ontology (GO) and Kyoto Encyclopedia of Genes and Genomes (KEGG) pathway enrichment analysis of DEGs. (**A**) GO term classification. (**B**) Top 20 significantly enriched GO terms (Q-values ≤ 0.05). (**C**) KEGG pathway classification. (**D**) Top 20 significantly enriched KEGG pathways (Q-values ≤ 0.05).

To investigate the biological response of the midgut to pyriproxyfen, we performed Gene Ontology (GO) enrichment analysis on the DEGs. Results showed that DEGs focused on molecular functions, cellular components, and biological processes (Figure 2A). The top 20 enriched GO terms comprised fourteen molecular function categories, three biological process categories, and three cellular component categories (Figure 2B).

KEGG pathway analysis of the DEGs identified significant enrichment in metabolic pathways categorized into cellular processes, environmental information processing, genetic information processing, metabolism, and organismal systems. These pathways encompassed 33 subcategories, including cell growth and death, cell motility, cellular community-eukaryotes and so on (Figure 2C). Among the 2059 identified DEGs, 971 genes were annotated with KO numbers and assigned to 248 pathways. Thirteen KEGG pathways showed significant enrichment, including ribosome, oxidative phosphorylation, and leucine and isoleucine degradation (Figure 2D).

### 3.3. Modulation of Chitin and Sugar Metabolism-Related Genes by Pyriproxyfen

Transcriptomic analysis of the midgut revealed significant changes in twelve chitin-related genes at 24 h post-exposure (Table 2). Specifically, nine genes were downregulated, while three were upregulated. Notably, key genes encoding chitin deacetylase (*CDA*) and chitin synthase (*CHS*), which are crucial for chitin synthesis and modification, were significantly inhibited.

In contrast to the downregulation observed in chitin genes, carbohydrate metabolism appeared largely activated. Of the thirty DEGs related to sugar metabolism, twenty-two (73.33%) were upregulated, while only eight were downregulated (Table 3).

### 3.4. Spatiotemporal Expression of Chitin-Related Genes Following Pyriproxyfen Exposure

Chitin is an important structural substance that makes up the cuticle and silk glands of silkworms, and it is also a component of the midgut peritrophic membrane [44,45]. To characterize the systemic effects associated with pyriproxyfen, we analyzed the spatiotemporal expression profiles of nine chitin-related genes (*CDA1*, *CDA2*, *ChsA*, *ChsB*, *Mucin-2-X1*, *Mucin-2-X2*, *Cht10*, *BMSK0001934*, and *BMSK0008490*) across five key tissues: the epidermis, anterior silk gland (ASG), middle silk gland (MSG), posterior silk gland (PSG), and midgut.

#### 3.4.1. Transcriptional Response of Chitin-Related Genes in the Epidermis

**Figure 3:**
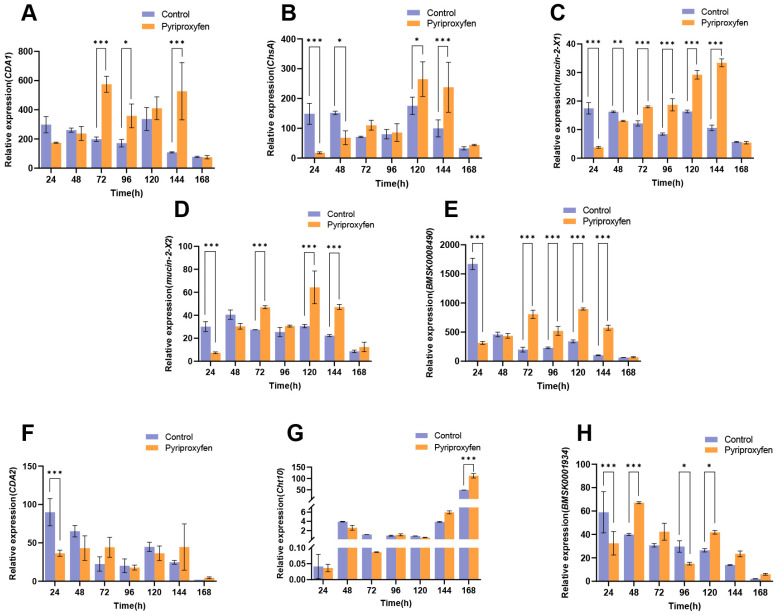
Transcriptional profiles of chitin-related genes in the epidermis following pyriproxyfen exposure. (**A**) *CDA1*; (**B**) *ChsA*; (**C**) *Mucin-2-X1*; (**D**) *Mucin-2-X2*; (**E**) *BMSK0008490*; (**F**) *CDA2*; (**G**) *Cht10*; (**H**) *BMSK0001934*. Data are presented as mean ± SD (n = 3). Significant differences are indicated by * *p* < 0.05, ** *p* < 0.01, and *** *p* < 0.001.

In the epidermis, most chitin-related genes (*CDA1*, *CDA2*, *ChsA*, *Mucin-2-X1*, *Mucin-2-X2*, *Cht10*, *BMSK0001934*, and *BMSK0008490*) exhibited a biphasic response characterized by initial downregulation followed by upregulation. Specifically, *CDA1*, *ChsA*, *Mucin-2-X1*, *Mucin-2-X2*, and *BMSK0008490* exhibited an identical variation trend. They were downregulated between 24 and 48 h but upregulated from 72 h to 144 h, and showed no significant difference at 168 h (Figure 3A–E). *CDA2* gene showed significant downregulation only at 24 h (*p* = 0.0001), followed by non-significant fluctuations (Figure 3F). Compared with other genes, the upregulation of the *Cht10* gene showed a lagging phenomenon after pyriproxyfen treatment. It was persistently suppressed at 24, 48, 72, and 120 h, with 0.89-fold, 0.19-fold, 0.05-fold, and 0.47-fold of the control group respectively. Although slight upward trends were observed at 96 h and 144 h, a significant upregulation was not achieved until 168 h, reaching 2.29-fold that of the control (*p* < 0.001) (Figure 3G). Interestingly, the *BMSK0001934* gene is specifically expressed in the epidermis of silkworms. Its transcriptional level displayed a fluctuating pattern, with significant downregulation at 24 h (*p* < 0.001) and 96 h (*p* = 0.0394), interspersed with upregulation at 48 h (*p* < 0.001) and 120 h (*p* = 0.0267) (Figure 3H). In summary, following pyriproxyfen treatment, the transcriptional levels of chitin-related genes in the epidermis generally exhibited a biphasic trend of initial downregulation followed by upregulation.

#### 3.4.2. Transcriptional Response of Chitin-Related Genes in the ASG

**Figure 4:**
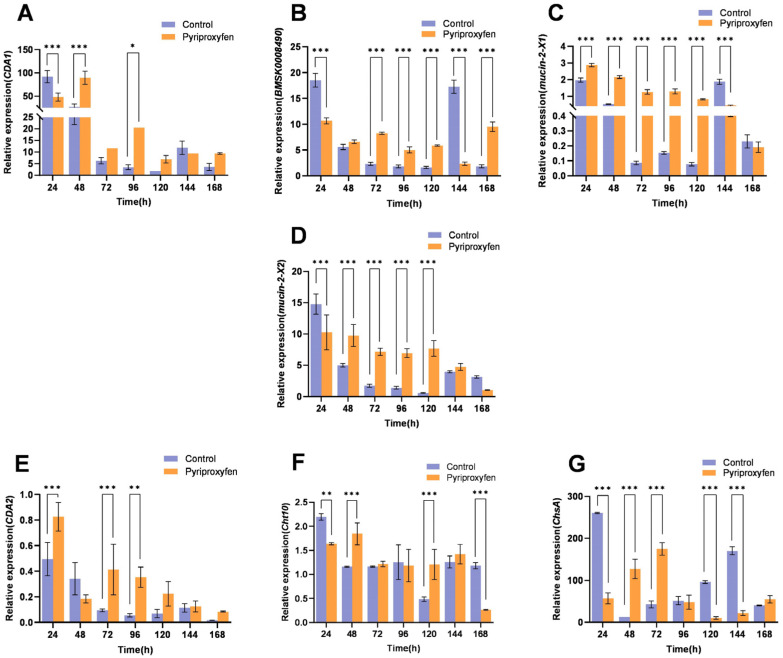
Transcriptional profiles of chitin-related genes in the ASG following pyriproxyfen exposure. (**A**) *CDA1*; (**B**) *BMSK0008490*; (**C**) *Mucin-2-X1*; (**D**) *Mucin-2-X2*; (**E**) *CDA2*; (**F**) *Cht10*; (**G**) *ChsA*. Data are presented as mean ± SD (n = 3). Significant differences are indicated by * *p* < 0.05, ** *p* < 0.01, and *** *p* < 0.001.

In the ASG, *CDA1* and *BMSK0008490* exhibited identical transcriptional patterns. Both genes were downregulated at 24 h, upregulated from 48 h to 120 h, transiently downregulated at 144 h, and upregulated again at 168 h (Figure 4A,B). In contrast to the trend observed in the epidermis, *Mucin-2-X1* gene in the ASG was upregulated during the early-to-mid stages (24–120 h) before being downregulated at 144 h to 168 h (Figure 3C and Figure 4C). The *Mucin-2-X2* gene was significantly downregulated at 24 h (*p* = 0.0001). Subsequently, it showed significant upregulation from 48 h to 120 h, with fold changes of 1.95, 4.11, 4.93, and 12.97, respectively (*p* < 0.001) (Figure 4D). The transcriptional levels of the *CDA2* gene were upregulated at most time points, with significant increases observed at 24 h (*p* = 0.0005), 72 h (*p* = 0.0007), and 96 h (*p* = 0.0017), except for a downregulation at 48 h (*p* = 0.2090) (Figure 4E). The *Cht10* gene generally showed a trend of first downregulation, then upregulation, and finally downregulation again. After a significant downregulation at 24 h (*p* = 0.0054), it shifted to upregulation at 48 h (*p* = 0.0006) and was significantly downregulated again at 168 h (*p* < 0.001) (Figure 4F). The *ChsA* gene displayed marked fluctuations. It was significantly downregulated at 24 h (*p* < 0.001), 120 h (*p* < 0.001), and 144 h (*p* < 0.001), but exhibited sharp, significant upregulation at 48 h (9.72-fold) (*p* < 0.001) and 72 h (4.08-fold) (*p* < 0.001) (Figure 4G). In summary, with the exception of *Mucin-2-X1* and *CDA2*, which were significantly upregulated as early as 24 h, chitin-related genes in the ASG generally exhibited an initial downregulation followed by upregulation in response to pyriproxyfen.

#### 3.4.3. Transcriptional Response of Chitin-Related Genes in the MSG

**Figure 5:**
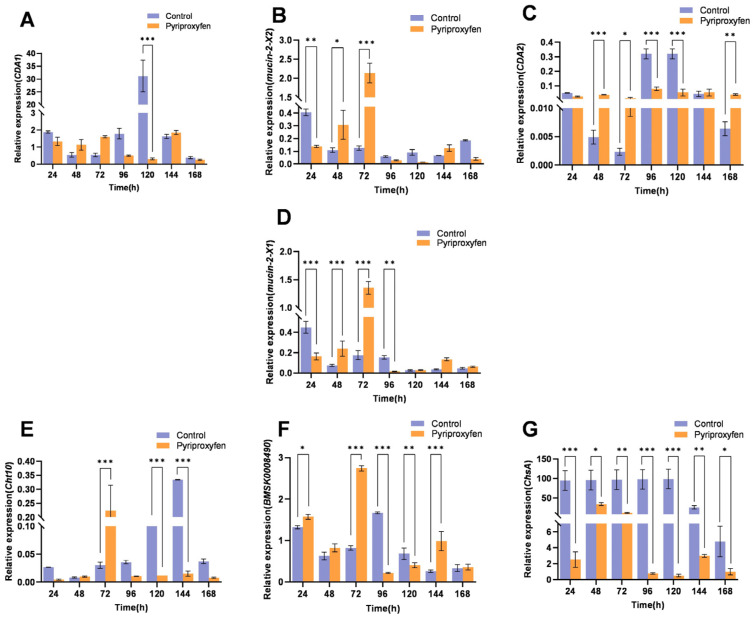
Transcriptional profiles of chitin-related genes in the MSG following pyriproxyfen exposure. (**A**) *CDA1*; (**B**) *Mucin-2-X2*; (**C**) *CDA2*; (**D**) *Mucin-2-X1*; (**E**) *Cht10*; (**F**) *BMSK0008490*; (**G**) *ChsA*. Data are presented as mean ± SD (n = 3). Significant differences are indicated by * *p* < 0.05, ** *p* < 0.01, and *** *p* < 0.001.

In the MSG, *CDA1* and *Mucin-2-X2* exhibited synchronized expression patterns characterized by downregulation at 24, 96, 120, and 168 h, interspersed with upregulation at 48, 72, and 144 h (Figure 5A,B). Similarly, *CDA2* and *Mucin-2-X1* exhibited the same variation pattern. They were downregulated at 24 h, 96 h, and 120 h, and upregulated at 48 h, 72 h, 144 h, and 168 h (Figure 5C,D). The transcription level of the *Cht10* gene fluctuated, showing initial suppression followed by a sharp peak at 72 h (7.43-fold, *p* < 0.001) before declining again after 96 h (Figure 5E). *BMSK0008490* generally presented a trend of first upregulation and then downregulation. It was upregulated at 24–72 h and 144 h, but significantly downregulated at 96 h (*p* < 0.001) and 120 h (*p* = 0.0064) (Figure 5F). Notably, *ChsA* transcription was consistently and significantly suppressed throughout the entire post-treatment period (24–168 h). The fold changes relative to the control group were 0.03 (*p* < 0.001), 0.36 (*p* = 0.0207), 0.12 (*p* = 0.0016), 0.01 (*p* < 0.001), 0.01 (*p* < 0.001), 0.12 (*p* = 0.0016), and 0.21 (*p* = 0.0104), respectively (Figure 5G). In summary, with the exception of *ChsA* and *BMSK0008490*, chitin-related genes in the MSG generally exhibited a biphasic trend of initial downregulation followed by upregulation.

#### 3.4.4. Transcriptional Response of Chitin-Related Genes in the PSG

**Figure 6:**
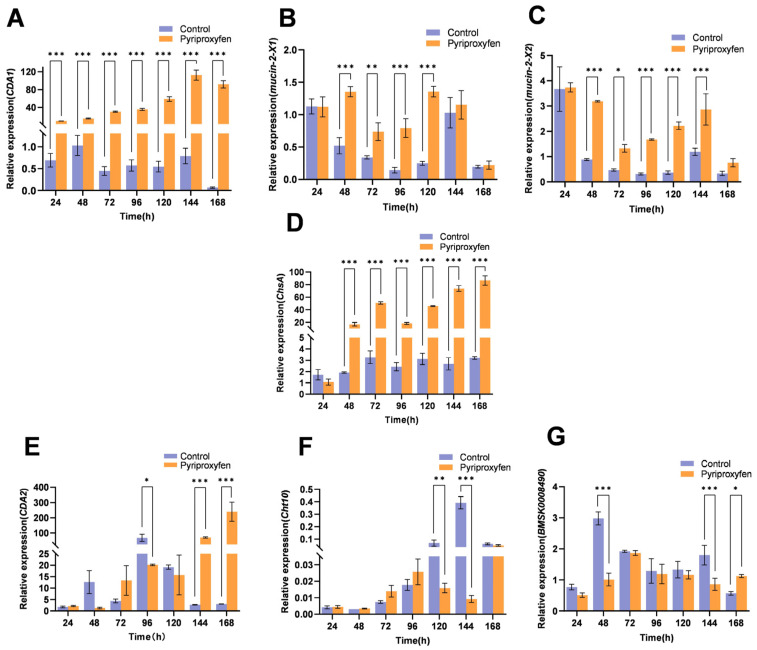
Transcriptional profiles of chitin-related genes in the PSG following pyriproxyfen exposure. (**A**) *CDA1*; (**B**) *Mucin-2-X1*; (**C**) *Mucin-2-X2*; (**D**) *ChsA*; (**E**) *CDA2*; (**F**) *Cht10*; (**G**) *BMSK0008490*. Data are presented as mean ± SD (n = 3). Significant differences are denoted by * *p* < 0.05, ** *p* < 0.01, and *** *p* < 0.001.

In the PSG, chitin-related genes exhibited divergent response patterns. *CDA1*, *Mucin-2-X1*, and *Mucin-2-X2* were consistently upregulated from 24 h to 168 h (Figure 6A–C). Strikingly, the response of *ChsA* in the PSG was opposite to that in the MSG. After being downregulated at 24 h, *ChsA* was significantly upregulated from 48 h to 168 h, with fold increases of 8.73, 15.59, 7.42, 14.73, 27.51, and 26.95, respectively (*p* < 0.001) (Figure 6D). *CDA2* gene expression fluctuated significantly; it showed non-significant changes at early stages (upregulation at 24 h and 72 h, downregulation at 48 h) and downregulation at 96–120 h. However, it surged dramatically from 144 h to 168 h, reaching 26.34-fold (*p* = 0.0005) and 79.41-fold (*p* < 0.001), respectively (Figure 6E). *Cht10* showed non-significant upregulation from 24 h to 96 h, followed by significant downregulation between 120 h (*p* = 0.0015) and 144 h (*p* < 0.001) (Figure 6F). *BMSK0008490* remained downregulated from 24 h to 144 h, with significant suppression at 48 h (*p* < 0.001) and 144 h (*p* < 0.001), before rebounding to significant upregulation at 168 h (*p* = 0.0177) (Figure 6G).

#### 3.4.5. Transcriptional Response of Chitin-Related Genes in the Midgut

**Figure 7:**
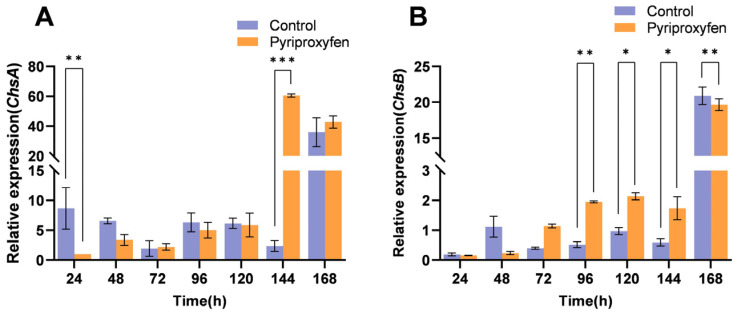
Transcriptional profiles of chitin-related genes in the midgut following pyriproxyfen exposure. (**A**) *ChsA*; (**B**) *ChsB*. Data are presented as mean ± SD (n = 3). Significant differences are denoted by * *p* < 0.05, ** *p* < 0.01, and *** *p* < 0.001.

In the midgut, *ChsA* gene generally exhibited a trend of initial downregulation followed by upregulation, similar to the pattern observed in the epidermis. It was downregulated at 24 h. Subsequently, the suppression attenuated, shifting to upregulation from 144 h to 168 h. Notably, the upregulation at 144 h reached a significant peak of 25.77-fold compared to the control (*p* < 0.001) (Figure 7A). Additionally, the *ChsB* gene was found to be specifically expressed in the midgut, with no detectable expression in the epidermis or silk glands. Its transcriptional level exhibited non-significant downregulation at 24 h and 48 h. This was followed by significant upregulation from 72 h to 144 h, with increases of 2.86-fold (*p* = 0.2468), 3.77-fold (*p* = 0.0020), 2.20-fold (*p* = 0.0148), and 2.93-fold (*p* = 0.0178), respectively, followed by significant downregulation at 168 h (*p* = 0.0085) (Figure 7B).

A comprehensive summary of the expression trends for all these chitin-related genes in different key tissues is presented in Table 4.

### 3.5. Transcriptional Response of Trehalose-Related Genes Following Pyriproxyfen Exposure

**Figure 8:**
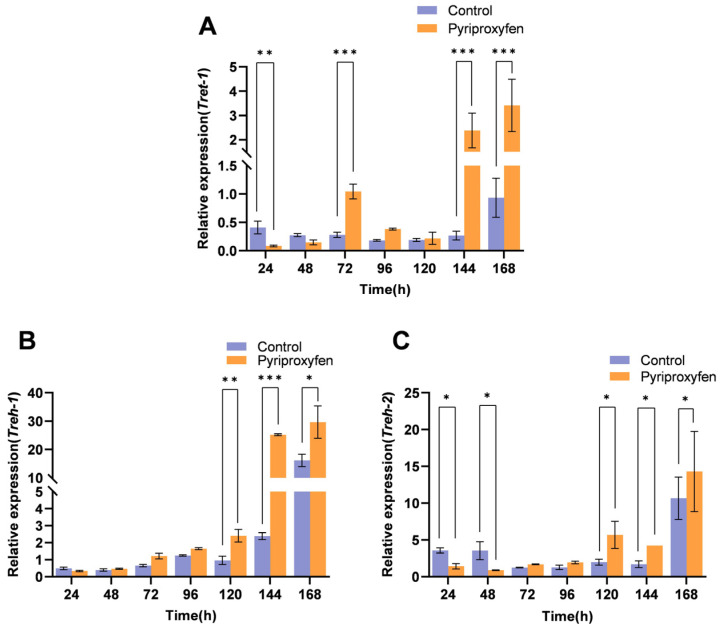
Transcriptional profiles of trehalose-related genes in the midgut following pyriproxyfen exposure. (**A**) *Tret-1*; (**B**) *Treh-1*; (**C**) *Treh-2*. Data are presented as mean ± SD (n = 3). Significant differences are denoted by * *p* < 0.05, ** *p* < 0.01, and *** *p* < 0.001.

To investigate the impact of pyriproxyfen on trehalose metabolism, we analyzed the spatiotemporal expression of *Treh-1*, *Treh-2*, and *Tret-1* in the midgut. All three genes exhibited a general biphasic trend of initial downregulation followed by upregulation. At 24 h post-exposure, all three genes were suppressed. *Treh-1* began to recover and upregulate by 48 h (*p* = 0.0929), whereas *Tret-1* and *Treh-2* started to upregulate at 72 h. By 144 h and 168 h, *Tret-1* levels in the treatment group were significantly elevated to 8.88-fold (*p* < 0.001) and 3.65-fold (*p* < 0.001) relative to controls, respectively (Figure 8A). Similarly, *Treh-1* reached peak levels of 10.55-fold (*p* < 0.001) and 1.83-fold (*p* = 0.0114) at these time points (Figure 8B). *Treh-2* remained downregulated at 24–48 h but sustained upregulation from 72 h to 168 h (Figure 8C).

## 4. Discussion

Our previous work demonstrated that the pesticide pyriproxyfen is associated with severe damage to silk glands and failure of metamorphosis in silkworms [38,40]. Interestingly, both physiological processes are critically dependent on chitin metabolism and its upstream substrate, trehalose. Chitin is a fundamental structural component of the insect exoskeleton, protecting against mechanical damage and facilitating molting [46]. In silkworms, chitin is also indispensable for the silk gland, interacting with cuticular proteins to form the cuticular intima of the spinning duct [44]. However, its transcriptional regulation and the involved gene network remain to be characterized in silkworms. Given that trehalose is the essential substrate for chitin synthesis [47], and the midgut functions as the primary site for both trehalose utilization and peritrophic membrane synthesis, we focused on characterizing the transcriptional landscape associated with this metabolic interplay in this tissue. Accordingly, we performed transcriptome profiling of the midgut 24 h post-exposure, identifying 2059 DEGs (1046 upregulated and 1013 downregulated) (Figure 1B). Subsequent spatiotemporal analysis of key chitin-related genes (*CDA1*, *CDA2*, *ChsA*, *ChsB*, *Mucin-2-X1*, *Mucin-2-X2*, *Cht10*, *BMSK0001934*, and *BMSK0008490*) revealed that, despite tissue-specific variations, these genes generally exhibited a biphasic expression pattern: initial transcriptional suppression followed by compensatory upregulation. This dynamic response is similar to the findings in *Locusta migratoria manilensis* and *Oxya chinensis* to flufenoxuron [48], where synthase genes exhibited a similar pattern of compensatory upregulation after an initial downregulation.

A pivotal finding of this study is the tissue-specific dysregulation of chitin metabolism genes by pyriproxyfen. CDA is a pivotal enzyme in chitin metabolism, responsible for deacetylating chitin into chitosan [49,50]. Insect CDAs are categorized into five groups (Groups I–V) based on sequence homology and domain architecture [49]. Among them, Group I members (*CDA1* and *CDA2*) are widely recognized for their association with molting and pupation in lepidopteran insects. Numerous functional studies have confirmed that silencing the expression of genes such as *CDA1* disrupts the development in pests such as *Choristoneura fumiferana*, *Mythimna separata*, *Tuta absoluta*, and *Tribolium castaneum*, causing abnormal molting and increased mortality [46,51,52,53]. Additionally, loss of *CDA* function in *Tribolium castaneum* results in severe cuticular and locomotor abnormalities [54]. In contrast, silencing of *BmCDA1* and *BmCDA2* in silkworm causes only pupation delay without lethal phenotypes [49], suggesting species-specific functional redundancy or divergence. However, pyriproxyfen exposure was associated with significant tissue-specific alterations in *CDA1* and *CDA2* expression. Notably, *CDA1* exhibited continuous and consistent upregulation in the PSG (Figure 6A). The PSG is the core site for fibroin synthesis, a function heavily dependent on the structural integrity of the chitin-protein intima lining its lumen [40,55]. We hypothesize that the aberrant overexpression of *CDA1* may represent a compensatory response to the intimal damage observed following pyriproxyfen exposure. However, this dysregulated repair attempt appears maladaptive and coincides with persistent glandular dysfunction, aligning with the silk-spinning disorders we previously observed [39,40]. Thus, the transcriptional dysregulation of *CDA1* and its associated pathway emerges as the most prominent correlate in our dataset, highlighting it as a key candidate molecular target in the PSG.

This disruption extends to the synthetic arm of chitin metabolism. Chitin synthase is the key rate-limiting enzyme in chitin biosynthesis, among which *ChsA* and *ChsB* usually have distinct tissue-specific functional divisions in insects. As a critical isoenzyme, *ChsA* is essential for survival; its loss leads to fatal molting defects, making it a prime target for insecticides [18,56]. Our results reveal a complex transcriptional outcome of pyriproxyfen exposure: rather than uniform suppression, it is associated with a striking spatiotemporal dichotomy. In the MSG, *ChsA* transcription was consistently and significantly suppressed (24–168 h) (Figure 5 G). This suppression is correlated with altered chitin deposition in the MSG cuticular intima, a structure vital for sericin secretion [57,58], which is consistent with the observed silk gland atrophy. Intriguingly, *ChsA* in the PSG was significantly upregulated during the same period (48–168 h) (Figure 6D). This contrasting expression pattern provides transcriptional evidence for a compensatory or dysregulated feedback response following pyriproxyfen exposure.

We hypothesize that following pyriproxyfen exposure, the observed alterations in the PSG coincide with a stress-induced upregulation of chitin synthesis that appears chaotic and ultimately ineffective. In this scenario, the failure to form normal structures would be expected to result in functional collapse.

Furthermore, this study is the first to report the regulation of the midgut-specific gene *ChsB* by pyriproxyfen. Unlike the dramatic fluctuations of *ChsA* in the silk gland, *ChsB* in the midgut exhibits a milder yet more persistent pattern of initial downregulation followed by upregulation (Figure 7B). This dynamic is consistent with the midgut’s dual role in detoxification and nutrient absorption. The early downregulation of *ChsB* is consistent with an energetic trade-off, in which transcriptional resources may be diverted from anabolic processes like chitin synthesis. Conversely, the significant upregulation in later stages (72–144 h) may coincide with or facilitate repair processes addressing potential damage to the peritrophic membrane, whose integrity relies on *ChsB*-mediated chitin synthesis [16,17]. This phenomenon mirrors observations in insects treated with chitin synthesis inhibitors, where abnormal elevation of chitinase expression fails to prevent molting failure [48].

Collectively, the differential regulation of *ChsA* and *ChsB* correlates with the multilevel physiological disruption associated with pyriproxyfen. The coordinated, multi-tissue disruption of the chitin synthesis pathway emerges as a central correlate of the insecticide’s high potency.

Beyond core enzymes, the response of structural components reveals novel insights. Although *Mucin* family genes are known to regulate insect tracheal and embryonic development [29,30,59], this study provides the first characterization of *Mucin-2-X1* and *Mucin-2-X2* under pesticide stress. Their complex, tissue-specific expression patterns rule out a generic stress response and are consistent with tissue-specific functional demands. For instance, their modulation in tissues critical for barrier function, such as the epidermis and midgut, points to a potential role in maintaining tissue integrity. Their expression in the silk gland, a tissue dedicated to secretion and ductal transport, similarly suggests a tissue-specific function. This functional diversification echoes findings in *Locusta migratoria* [59], and underscores the limitation of viewing the *Mucin* family as a functional monolith. Future research should focus on the specific roles of these subtypes as potential molecular nodes linking hormonal signaling to tissue remodeling.

The observed changes are consistent with a systemic metabolic shift. Chitin biosynthesis is energetically expensive and strictly dependent on trehalose metabolism, forming a tightly coupled physiological module [60,61,62]. In this study, we observed that following pyriproxyfen exposure, trehalose-related genes (*Treh-1*, *Treh-2*, and *Tret-1*) exhibited a biphasic expression profile characterized by initial downregulation followed by upregulation, closely paralleling the pattern observed in most chitin genes (Figure 8). This coordinated pattern suggests a coupled transcriptional response to pyriproxyfen stress. The initial transcriptional suppression likely reflects an active energy trade-off, diverting limited resources from costly anabolism, such as chitin synthesis, toward immediate survival mechanisms like detoxification. This type of resource reallocation under xenobiotic stress is a conserved strategy in insects, mediated by energy-sensing pathways. For example, the AMPK—CncC pathway explicitly regulates trade-offs between detoxification and other energy-demanding processes [63]. Similar metabolic prioritization has been observed in *Nilaparvata lugens* under pathogenic stress [31].

Conversely, the global transcriptional upregulation observed in the later stages of stress is consistent with the possibility of a compensatory transcriptional response. This upregulation of key genes in the trehalose and chitin synthesis pathways occurs subsequent to the initial stress phase and is focused on tissues critical for development, such as the silk gland and epidermis. This compensatory surge is consistent with findings in locusts treated with flufenoxuron [48]. However, the ultimate failure of these silkworms to complete metamorphosis or spin silk indicates that this compensation is insufficient or functionally ineffective under continuous pyriproxyfen interference. Whether this failure stems from translational blocks, enzymatic inactivity, or defective chitin deposition remains a key question for future investigation.

Nevertheless, it should be noted that a limitation of this study is the reliance on transcriptional data. While we observed strong correlations between gene expression changes and the physiological defects reported in our previous works, functional validation (e.g., via RNAi or gene editing) is required to definitively establish causality between specific gene dysregulation and phenotypic outcomes.

## 5. Conclusions

This study provides a comprehensive transcriptional characterization of pyriproxyfen toxicity. In summary, our findings reveal that the toxicity of pyriproxyfen is associated with its capacity to disrupt the critical coordination between trehalose metabolism and chitin biosynthesis in silkworms. The key outcomes are tissue-specific gene expression disorders and a consequent failure of physiological homeostasis. These results highlight the vulnerability of beneficial insects, such as the silkworm, to metabolic disruptors by triggering complex, tissue-specific transcriptional alterations in metabolic genes. To mitigate such off-target effects, practical measures could include establishing buffer zones around sericulture areas or developing next-generation insecticides with greater target specificity to protect non-target species.

## Figures and Tables

**Table 1:** Primer sequences used in this study.

Gene Name	Gene ID	Primer Sequence (5′-3′)
*Actin3*	100145915	F: CTGCGTCTGGACTTGGC R: CGAGGGAGCTGCTGGAT
*CDA1*	732885	F: TCAGTTGTGCGACGGTAGAC R: GGTGACGCTGTTCTCTAGGC
*CDA2*	732885	F: ACAAGATGACGACGGAGCTG R: ACCTGACCTAGTGCACCTGA
*ChsA*	100884166	F: ACAAGAGGCTCGCATAGCAG R: CCAGACCACGTGAAGCTGAT
*ChsB*	101744621	F: ATCCAGTCGGCTCAGGTTTC R: TCGTCCATCAGAGCCTTTCC
*Cht10*	692403	F: GAGGGTTATGCGGTTCACCA R: GCATTTGAGTCGCACGATCC
*Mucin-2-X1* (*CHIA*)	101743162	F: GAATTACGCTTTCCGCCGTC R: GCTCGTGCTTGGAACCTACT
*Mucin-2-X2* (*BMSK0004667*)	101743162	F: GCCTGAGGTGGACATCGAAA R: ACGAACCTATTAGGGGGCCT
*BMSK0001934*	114250954	F: TTTCCGTCTCACCACCGAAG R: TCGTGGCTTCTCGAGTTTGT
*BMSK0008490*	733010	F: AGAGTCCTTCAAATGCCCCG R: TGGAATCTGTGGCGTCGAAA
*Treh-2*	692454	F: GTGTCGTTGCTGATCGTAGCA R: GCCCGTGGCAGTAAATCATAC
*Treh-1*	693027	F: TAGCAACCTAGCTCCCCTGT R: TTCTGGAGGCCAAGCGTTAG
*Tret1*	100141437	F: GATCACGACACTCTTAGCACTA R: CCAACACATAGATAACGAGGCA
*Iim1*	100862783	F: GGTTGCCCAGTTAACCCTCA R: CCAGTCGCAAACCTGGATGA
*Cp-2*	101738492	F: TATGCTGGAACCACAAGCCT R: TGAGGCCAATCGCATTCTTCT
*Cdp1*	101745440	F: CATGCGACCTACCACCAAGA R: AACCTTTGTTGTTCCACGGC
*Wol*	101738398	F: ACGTATCCTGTTGTGGAGCG R: CCAACATGGGTGGCAGTCTT
*LOC101740802*	101740802	F: TAGCGGTCAACATGAACAAAGC R: GTCTCCGGGTTCTGTCTCAA
*Sec13*	101741617	F: GAGATACTTCATAAGTAACGCGCAA R: GAATGCCGCTCGTGTGTTAG
*Gd*	101735751	F: TGACCTCCCTGTCGGATACA R: GTGATGCAGCCGTTAAATCGT
*Pfk*	101745490	F: GGTCGCAACTGTGGTTATTTGG R: TTGGGAACAGGGTCTTCAGGT
*Minpp1*	101739930	F: GGCAAAGGTGAGCTATCCGA R: GCCGTGTAGCGAAACAGGAA
*Gk-2*	100141427	F: GAGTGATGTGCGGTTTGAGC R: ATGAGGGGACGAATGACTGG

**Table 2:** Expression levels of chitin-related genes in midgut following pyriproxyfen exposure.

Gene	Brief Description	Q-Value	Log_2_ (Treatment/Control)
*LOC101740647 (CDA)*	chitin binding	0.048283	−1.18
*LOC732885*	chitin binding	8.53 × 10^−5^	−1.99
*LOC114243151 (CHS)*	chitin synthase activity	0.001111	−1.71
*LOC101744560*	chitin binding	2.64 × 10^−8^	−3.14
*LOC101740330*	chitin binding	2.82 × 10^−5^	−2.17
*LOC101739926*	chitin binding	0.001605	−1.65
*LOC101743739*	chitin binding	0.009699	−1.40
*LOC101735751*	pupal chitin-based cuticle development	1.76 × 10^−5^	−2.27
*LOC100862783*	chitin binding	5.53 × 10^−8^	4.24
*LOC101738492*	chitin binding	4.25 × 10^−8^	4.22
*LOC101742268*	chitin-based cuticle development	0.002742	−1.13
*LOC101738097 (Mucin-2)*	chitin binding	0.000382	1.76

**Table 3:** Expression levels of sugar metabolism-related genes in midgut following pyriproxyfen exposure.

Gene	Brief Description	Q-Value	Log_2_ (Treatment/Control)
*LOC101744252*	response to trehalose	0.000394	−2.16
*LOC733033*	UDP-sugar diphosphatase activity	0.038280	−1.44
*LOC114252750*	carbohydrate transport	0.021491	1.53
*LOC692946*	carbohydrate binding	0.010693	−1.64
*LOC101740647*	carbohydrate metabolic process	0.001991	−1.18
*LOC732885*	carbohydrate metabolic process	0.001155	−1.99
*LOC100379324*	carbohydrate metabolic process	4.59 × 10^−6^	2.87
*LOC101744477*	carbohydrate metabolic process	0.012020	1.26
*LOC101744143*	carbohydrate metabolic process	0.003541	1.09
*LOC101742223*	carbohydrate metabolic process	0.012021	−1.26
*LOC692952*	carbohydrate metabolic process	0.001281	1.25
*LOC100145905*	carbohydrate metabolic process	0.007710	1.33
*LOC110386457*	carbohydrate metabolic process	0.016938	1.57
*LOC101737249*	carbohydrate metabolic process	3.85 × 10^−9^	−3.98
*LOC692629*	carbohydrate binding	0.047950	1.40
*LOC100307005*	carbohydrate metabolic process	0.000236	2.24
*LOC101736379*	carbohydrate metabolic process	0.013922	−1.60
*LOC101735491*	carbohydrate metabolic process	0.000376	2.17
*LOC100037426*	canonical glycolysis	0.001302	1.27
*LOC693076*	canonical glycolysis	0.040181	1.07
*LOC100462719*	regulation of gluconeogenesis	0.000124	1.75
*LOC101736985*	gluconeogenesis	0.004044	1.07
*LOC778462*	gluconeogenesis	0.002923	1.12
*LOC692968*	positive regulation of glycogen biosynthetic process	0.001883	1.19
*UGP2*	glycogen metabolic process	0.002421	1.15
*LOC101737567*	tricarboxylic acid cycle	0.042694	1.06
*LOC101735810*	tricarboxylic acid cycle	6.73 × 10^−5^	1.01
*LOC100101211*	glucose metabolic process	2.19 × 10^−5^	1.23
*LOC101740061*	glucose metabolic process	6.73 × 10^−5^	1.13

**Table 4:** Expression trends of chitin-related genes in different key tissues.

Gene Name	Epidermis	ASG	MSG	PSG	Midgut
*ChsA*	biphasic	biphasic	sustained suppression	delayed yet persistent upregulation	biphasic
*CDA1*	biphasic	biphasic	biphasic	persistent upregulation	——
*ChsB*	——	——	——	——	biphasic
*CDA2*	biphasic	biphasic	biphasic	biphasic	——
*Mucin-2-X1*	biphasic	biphasic	biphasic	persistent upregulation	——
*Mucin-2-X2*	biphasic	biphasic	biphasic	persistent upregulation	——
*Cht10*	biphasic	biphasic	biphasic	biphasic	——
*BMSK0008490*	biphasic	biphasic	biphasic	biphasic	——
*BMSK0001934*	biphasic	——	——	——	——

## Data Availability

The raw data supporting the conclusions of this article will be made available by the authors on request.
